# The Dual Roles of Activating Transcription Factor 3 (ATF3) in Inflammation, Apoptosis, Ferroptosis, and Pathogen Infection Responses

**DOI:** 10.3390/ijms25020824

**Published:** 2024-01-09

**Authors:** Shuang Liu, Zhangcheng Li, Shimei Lan, Huafang Hao, Ahmed Adel Baz, Xinmin Yan, Pengcheng Gao, Shengli Chen, Yuefeng Chu

**Affiliations:** 1 State Key Laboratory for Animal Disease Control and Prevention, College of Veterinary Medicine, Lanzhou University, Lanzhou Veterinary Research Institute, Chinese Academy of Agricultural Sciences, Lanzhou 730000, China; 2 Gansu Province Research Center for Basic Disciplines of Pathogen Biology, Lanzhou 730046, China; 3 Key Laboratory of Veterinary Etiological Biology, Key Laboratory of Ruminant Disease Prevention and Control (West), Ministry of Agricultural and Rural Affairs, Lanzhou 730046, China

**Keywords:** ATF3, inflammatory, apoptosis, ferroptosis, microorganism

## Abstract

Transcription factors are pivotal regulators in the cellular life process. Activating transcription factor 3 (ATF3), a member of the ATF/CREB (cAMP response element-binding protein) family, plays a crucial role as cells respond to various stresses and damage. As a transcription factor, ATF3 significantly influences signal transduction regulation, orchestrating a variety of signaling pathways, including apoptosis, ferroptosis, and cellular differentiation. In addition, ATF3 serves as an essential link between inflammation, oxidative stress, and immune responses. This review summarizes the recent advances in research on ATF3 activation and its role in regulating inflammatory responses, cell apoptosis, and ferroptosis while exploring the dual functions of ATF3 in these processes. Additionally, this article discusses the role of ATF3 in diseases related to pathogenic microbial infections. Our review may be helpful to better understand the role of ATF3 in cellular responses and disease progression, thus promoting advancements in clinical treatments for inflammation and oxidative stress-related diseases.

## 1. Introduction

Transcription factors (TFs) play a crucial role in the regulation of cellular function and the development of diseases due to their direct influence on gene expression, thus becoming a focal point of research in recent years. TFs regulate a variety of cellular physiological activities and immune responses by initiating or inhibiting the transcription of specific genes. Serving as key regulatory factors in the coordination of immune responses, TFs are essential for the activation of immune cells and their direct involvement in the production of inflammatory cytokines [1,2,3]. However, in the prolonged struggle between pathogens and hosts, some pathogenic microorganisms have evolved strategies to manipulate host TFs, thereby promoting their survival and replication within the host. Studies have elucidated that specific pathogenic organisms manipulate the activation of the NF-κB pathway as a survival strategy, which can effectively protect the host cell from apoptosis and allow bacterial survival within host cells [2]. Therefore, TFs are not only central to the host immune response but are also a vital strategy for some pathogens to establish infection. Increasing evidence suggests that TFs, such as STAT1, STAT2, JunB, CHOP, ATF3, and NF-κB, are involved in the interaction between host and pathogen and the regulation of host immune responses [2,4,5,6,7]. Thus, understanding the specific roles of TF in different infectious environments can provide insight for the development of targeted therapies to enhance host defense capability.

Activating transcription factor 3 (ATF3) is a stress-induced transcription factor that belongs to the activating transcription factor/cAMP response element-binding protein (ATF/CREB) family [8,9], whose members include ATF1, CREB, CREM, ATF2, ATF3, ATF4, ATF5, ATF6, ATF7, and B-ATF [10]. ATF3 regulates gene transcription by forming homodimers or heterodimers through its basic-region leucine zipper (bZIP) domain, thus modulating the biological functions of genes. ATF3 expression is relatively stable under normal physiological conditions, but changes in its expression are associated with various pathophysiological responses such as inflammation, oxidative stress, stress of the endoplasmic reticulum, and cell death [8,9,11]. ATF3 expression is induced by a variety of signals, including those initiated by cytokines, genotoxic agents, or physiological stressors [10]. ATF3 is upregulated under multiple stress conditions, regulating the interaction between cellular metabolism and immune and inflammatory responses, thus maintaining cellular homeostasis. Interestingly, unlike other members of the ATF family, increasing evidence suggests the involvement of ATF3 in the regulation of the host–pathogen interaction process [4,12,13,14,15,16,17]. Therefore, this review discusses the role of ATF3 in the regulation of cellular apoptosis, ferroptosis, and inflammatory responses, with a particular focus on the complex regulatory role of ATF3 in pathogen infections, and explains its bidirectional regulatory role in these processes.

## 2. Literature Screening

We conducted a literature search using the following search items on PubMed and Web of Science (all databases, topic): ATF3, ATF3 inflammation, ATF3 apoptosis, ATF3 ferroptosis, ATF3 virus, ATF3 bacteria, ATF3 fungi, and ATF3 parasite. References from all sources were reviewed to identify relevant articles. The gathered literature underwent a first round of screening, with exclusion criteria including duplicate articles, non-English manuscripts, and those without full-text availability. This was followed by a second round of screening, where articles were manually evaluated based on their titles, abstracts, or full texts to assess the relevance of ATF3 to inflammation, cell apoptosis, cell ferroptosis, and pathogenic microbes. During this screening process, a ‘citation within a citation’ or snowballing method was also employed to discover additional papers that may have been overlooked in the initial literature screening process. High-quality research findings published in authoritative journals were summarized and given priority in citations in the review.

## 3. Mechanisms of ATF3 Induction under Stressful Physiological Conditions

ATF3 shares the same binding site, 5′-TGACGTCA-3′, found in other transcription factors of the ATF/CREB family [10]. They interact with target DNA by binding to the entire region within the bZIP domain [8,10]. Several genes have been identified to possess this recognition sequence, including *Nrf2*, *JunD*, *c-Jun*, and *IL-6* [18,19]. Intriguingly, some promoters of target genes regulated by ATF3 have binding sequences that differ from these common motifs (Table S1). ATF3 expression is relatively stable under normal physiological conditions; however, it rapidly changes in response to disturbances in the internal environment or external stimuli [20]. Changes in ATF3 expression are induced by inflammatory responses, cell death, cytokines, and cellular stresses (oxidative stress, DNA damage, or endoplasmic reticulum stress (ERS)). The specific induction mechanism may vary depending on the type of stress but usually involves the activation of stress-responsive kinases and upstream transcription factors, which then bind to the *ATF3* promoter and stimulate its transcription [21,22]. Due to its induction in response to various stress signals, ATF3 is considered a stress-induced gene [23,24,25], which participates in cellular growth, tissue remodeling, cytoskeletal reorganization, and inflammation [21]. NF-E2-related factor 2 (Nrf2) transcriptionally upregulates ATF3 expression in astrocytes, thus promoting antioxidant and cytoprotective functions [26]. Naringin (Nar) activates ATF3 and inhibits PINK1 transcription by suppressing ERS and mitochondrial autophagy-related genes, as well as the expression of downstream ERS proteins [27]. Studies have shown that erastin can induce upregulation of ATF3 expression, which then strongly binds to the *SLC7A11* promoter, promoting ferroptosis in cells [28]. ATF3 is also involved in the signaling pathways of the response to DNA damage. Following damage to DNA, the *ATF3* promoter can be activated by MEKK1, suggesting that the involvement of the MAPK pathway is likely in the induction of ATF3 after DNA damage [29]. Furthermore, studies have shown that the JNK/SAPK signaling pathway can also induce ATF3 expression [30,31]. In particular, in studies using hydrogen peroxide as a stress signal, ATF3 induction was almost entirely inhibited by the antioxidant agent N-acetyl-L-cysteine, indicating that the induction of ATF3 requires oxidative stress [32,33].

ATF3 interacts with the cAMP response element (CRE) sequences through its basic region and forms homodimers or heterodimers with other CREB family members through its bZip domain [21,34]. Heterodimers, such as those formed by ATF3 with C-jun, ATF2, and JunB, have been demonstrated to possess transcriptional activation capabilities, enhancing the transcriptional activity of downstream target genes [32,35,36]. Currently, it is widely accepted that ATF3 might occur through stabilizing inhibitory cofactors on the promoter, thereby suppressing the transcriptional activity of downstream genes [19,34]. ATF3 recruits histone deacetylase 1 (HDAC1) to promoters containing ATF3 binding sites. Subsequently, HDAC1 causes histone deacetylation, leading to chromatin condensation and transcriptional repression [19,37]. Histone acetylation is a crucial physiological process that opens the chromatin structure, allowing transcription factors to bind to gene promoters and activate transcription [19,38]. Additionally, the various transcripts of ATF3 may represent another potential mechanism for its transcriptional activation capabilities [37]. The splice variant of ATF3, ATF3ΔZip2, inhibits the formation of the histone acetyltransferase (HAT) complex, thereby indirectly preventing histone acetylation [19,39,40]. Interestingly, the transcriptional inhibitory function of the ATF3 transcription factor might also be achieved through its binding to specific combinations of cis-acting elements. The homodimers of ATF3 possess the ability to inhibit the transcription of promoters containing its binding sites [21,37]. Recent studies have identified two adjacent ATF3 binding sites on the *SLC7A11* promoter sequence, suggesting that binding to these two sites may facilitate the formation of ATF3 homodimers, thereby repressing gene transcription [28]. Notably, the *SLC7A11* promoter possesses BS-1/BS-2 sequences and C/EBP-ATF response elements, allowing binding using many activators from the C/EBP and ATF/CREB transcription factor families [28]. This also implies that ATF3 might inhibit the expression of SLC7A11 by competing with transcription activators/co-activators for promoter binding. A similar phenomenon has been observed in the transcriptional regulation of ATF3 itself [41]. Two adjacent ATF3 binding sites have been found in its own promoter, leading to transcriptional autorepression by ATF3 [41]. This indicates that the presence of multiple adjacent ATF3 binding sites within a promoter sequence could lead to the formation of homodimers, potentially resulting in the inhibition of transcription. However, further experimental data are required to substantiate this hypothesis.

## 4. Implications of PAMPs and PRRs Activation on the Expression of ATF3

The Toll-like receptor (TLR) family consists of pattern recognition receptors (PRRs) involved in the detection of pathogen-associated molecular patterns (PAMPs) [10,42]. TLR1, 2, 4, 5, and 6 are expressed on the cell surface membrane and recognize bacterial and fungal products, while TLR3, 7, 8, and 9 are located in intracellular endosomes, specializing in the detection of pathogen-associated nucleic acids [10,42]. Upon recognition of their ligands, TLRs initiate complex cell signaling pathways, conferring antiviral and antibacterial states to the cell and promoting the expression of inflammatory cytokines, chemokines, and co-stimulatory molecules, which are crucial for the activation of adaptive immune responses [10,42]. Studies have shown that murine bone marrow macrophages treated with multiple PAMPs recognized by TLRs such as LPS, pIC, CpG-ODN, pIC/CpG-ODN, and zymosan significantly increase ATF3 protein expression [43], indicating that ATF3 expression is induced by various TLR responses. According to this, ATF3 is a potential transcriptional regulator in the TLR signaling cascade in macrophages. Furthermore, TLR4 mediates ATF3 expression in RAW 264.7 cells infected with *Streptococcus pneumoniae* through the JNK/p38 pathway [17]. This further implies the significant role of ATF3 in the recognition of PAMPs and the activation response of PRRs.

## 5. Modulatory Role of ATF3 in Inflammatory Cytokine

As a pivotal hub in the network of cellular adaptive responses and a transcription factor regulating immune response genes, ATF3 is a key regulator of both local and systemic inflammation, aiding cells in responding to disruptions in internal homeostasis. This is due to its ability to positively or negatively modulate the functional status or bioactivities of immune and nonimmune cells. The role of ATF3 has been studied in different contexts. In macrophages, ATF3 is a crucial regulator of innate immune responses. It is a product of TLR signaling and modulates inflammation in lipopolysaccharide (LPS)-stimulated cells by returning to this pathway [43,44,45,46]. Monocyte chemotactic protein-1-induced protein 1 (MCPIP1) inhibits macrophage polarization of M1 and promotes polarization of M2 by regulating the ATF3-AP1S2 signaling pathway, limiting the expression of pro-inflammatory cytokines in monocytes from patients with active inflammatory bowel disease (IBD). ATF3 can bind to the *AP1S2* promoter and induce inflammation [47]. Postprandial triglyceride-rich lipoprotein (TGRL) lipolysis products (TL) induce the expression of pro-inflammatory factors in human brain microvascular endothelial cells by upregulating ATF3 through the activation of mitochondrial oxidative stress [48]. NF-κB regulates its expression by binding to the *IL-6* promoter [49,50], while ATF3 has been shown to modulate NF-κB-dependent transcription by altering the phosphorylation of IκBα [34]. In osteoarthritis (OA), ATF3 modulates phosphorylation of p65 by altering IκB phosphorylation, which regulates the NF-κB signaling pathway, thus regulating the expression of IL-6 in chondrocytes [51]. ATF3 mediates particulate matter (PM)-induced inflammatory cytokine expression through the NF-κB and AP-1 pathways [52]. After infection with *Streptococcus pneumoniae* (*S. pneumoniae*), ATF3 regulates GPB5 or forms a complex with JUN, thus promoting the production of inflammatory cytokines (TNF-α, IL-1β, IL-6, and IFN-γ) [4,17].

**Figure 1:**
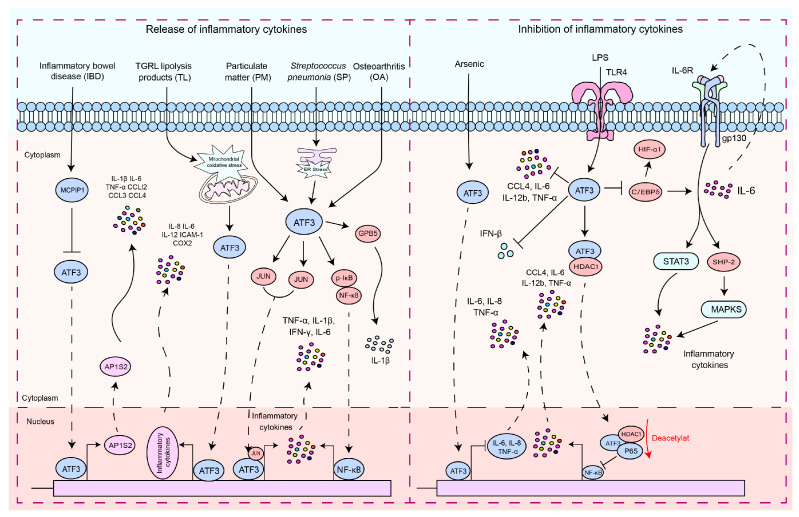
The signaling pathways by which ATF3 regulates the inflammatory response. These signaling pathways by which ATF3 regulates the inflammatory response can be divided into two types: (1) pro-inflammatory response—ATF3 increased the production of pro-inflammatory cytokines and chemokines by enhancing AP1S2 expression by binding to *AP1S2* promoters in inflammation of the intestine (IBD). *S. pneumoniae* (SP) stimulates the formation of an ATF3 complex with c-Jun, and this complex binds to cytokine promoters of cytokines (TNF-α, IL-1β, and IFN-γ), resulting in increased cytokine production. During an infection, lung macrophages quickly phagocytose invasive *S. pneumoniae*, resulting in ER stress and ATF3 activation. ATF3 then promotes GBP5 activation, triggering IL-1β secretion. TGRL lipolysis products (TL) potentiate ROS in mitochondria, activating mitochondrial oxidative stress and ATF3 signaling. Furthermore, ATF3 regulates TL-induced inflammation. ATF3 may positively regulate IL-6 expression in osteoarthritis (OA) chondrocytes through modulation of NF-κB-dependent transcription by modifying IκB phosphorylation. ATF3 may heterodimerize with c-JUN and activate IL-6 transcription in HBE cells induced by PM. (2) Anti-inflammatory response, including regulation of the ATF3/HDAC1/NF-κB axis and ATF3/C-EBPδ axis—ATF3 inhibits the production of inflammatory cytokines by suppressing C/EBPδ. ATF3 inhibits the production of pro-inflammatory cytokines by recruiting HDAC1 into the ATF3/p65 complex and facilitating the deacetylation of p65. ATF3 acted as a transcriptional repressor and regulated IFN-β. LPS activates ATF3 by stimulating TLRs, thus inhibiting the production of inflammatory cytokines. Solid arrows indicate promotion, dashed arrows denote translocation, horizontal arrows represent inhibition, and red arrows signify the processes of modification undergone.

ATF3 plays a dual role in the regulation of inflammatory responses (Figure 1). In endotoxin-stimulated monocytes, the stress associated with reactive oxygen species (ROS) leads to the induction of ATF3 expression and inhibits IL-6 production, making mice highly susceptible to secondary bacterial and fungal infections [53]. Negative regulation of transcription by ATF3 may be achieved through the inhibition of CCAAT/enhancer binding protein δ (C/EBPδ), a positive regulator of cytokine gene induction [54,55]. C/EBPδ enhances the expression of IL-6 [54], and in turn, IL-6, through activation of IL-6R, can activate the STAT3, SHP-2, and MAPK pathways, thus promoting cytokine secretion [54,56,57]. It is reported that ATF3 recruits HDAC1 to the ATF3/p65 complex and promotes the deacetylation of p65 to inhibit the production of pro-inflammatory cytokines [58]. The role of ATF3 in macrophages is not limited to the regulation of pro-inflammatory cytokines. It is also essential for regulating the production of interferon (IFN)-β downstream of innate immune receptors. ATF3 acts as a transcriptional repressor by binding to regulatory sites on the *Ifnb1* promoter [59]. The expression of macrophage inflammatory protein 1β (CCL4) leads to the onset of inflammatory diseases [44,60]. ATF3 inhibits the expression of CCL4 in mouse macrophages induced by LPS [44]. ATF3 negatively regulates the gene expression of IL-6 and IL-12 in macrophages by altering the structure of chromatin [19,43]. LPS activation of TLR4 induces ATF3 expression, which in turn suppresses the expression of various inflammatory genes induced by TLR4 signaling, including IL-6, IL-12b, and TNF-α [19,20]. Arsenic exposure-induced upregulation of ATF3 inhibits inflammatory responses by suppressing the production of cytokines such as IL-6, IL-8, and TNF-α [61]. ATF3, as a high-density lipoprotein (HDL)-inducible target gene in macrophages, has been reported to downregulate the expression of TLR-induced pro-inflammatory cytokines [62]. This suggests that ATF3 can negatively regulate the transcription of pro-inflammatory cytokines. By inhibiting the expression of pro-inflammatory genes, ATF3 can prevent an overactive immune response and potentially serve as a key regulator in the fight against invading pathogens and inflammatory diseases.

## 6. The Role of ATF3 in the Regulation of Cell Death

### 6.1. The Regulation of Apoptosis

**Figure 2:**
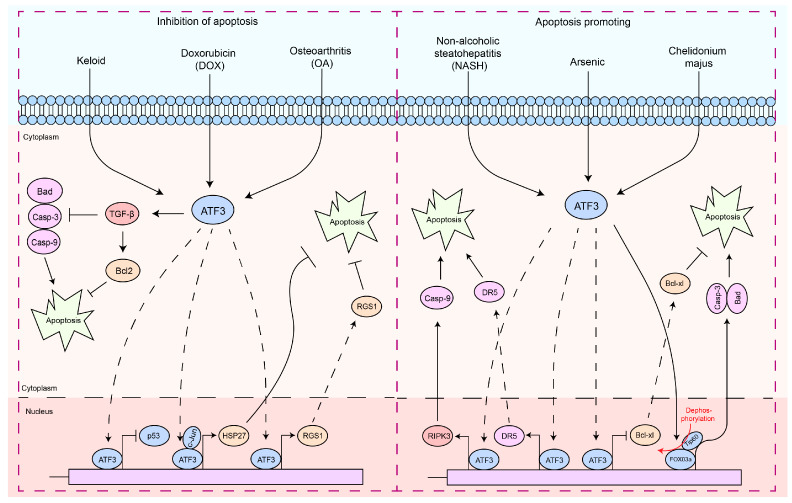
The functions of ATF3 in the response to apoptosis. These signaling pathways by which ATF3 regulates the apoptosis response can be divided into two types: (1) antiapoptotic response—ATF3 can regulate apoptosis of cells by upregulating the expression of the anti-apoptotic gene (*HSP27*, *RGS1*, and *Bcl2*) by binding to promoters, preventing p53 expression, which inhibits Caspases-3/9 activities. In addition, ATF3 inhibits Bad expression via TGF-β. (2) Pro-apoptosis response—ATF3 triggers the apoptotic pathway by upregulating RIPK3, DR5, and Caspase-9 by binding to their promoter, while concurrently inhibiting BCL-XL by binding to its promoter. Furthermore, ATF3 can also promote Caspase-3 and Bad transcription by activating FOXO3a, thereby regulating cell apoptosis. Solid arrows indicate promotion, dashed arrows denote translocation, horizontal arrows represent inhibition, and red arrows signify the processes of modification undergone.

Apoptosis is a finely regulated process of programmed cell death that plays a crucial role in the maintenance of physiological functions in organisms, as well as in interactions between pathogens and hosts [63,64,65,66]. Apoptosis is a form of cell death by which the body maintains the homeostasis of the internal environment [5,67]. In addition, it plays an important role in the regulation of the immune system, especially in autoreactive immune cells [5,68,69,70]. In the interaction between pathogens and hosts, apoptosis can serve as a host defense mechanism to limit the replication and spread of pathogens [5,71,72]. In contrast, certain pathogens may exploit host defense mechanisms to evade host immune responses or induce excessive inflammation and tissue damage during infection [5,71,72]. Therefore, the diverse and complex functions of apoptosis are essential to understanding cellular behavior in physiological and pathological states. In particular, ATF3 induction appears to be consistently associated with cellular damage, as most of the signals that induce ATF3 also induce cellular injury [21,32,73]. Interestingly, ATF3 exhibits a dual role in the regulation of apoptosis (Figure 2). In cardiomyocytes, ATF3 effectively inhibits doxorubicin-induced apoptosis [74]. Similarly, adenovirus-mediated expression of ATF3 protects neuronal cells from apoptosis induced by mitogen-activated kinase kinase kinase 1 (MEKK1)–c-Jun N-terminal kinase (JNK) [36]. The combination of ATF3 and c-Jun induces the antiapoptotic factor Hsp27, which activates Akt directly or indirectly, potentially inhibiting apoptosis and inducing neurite outgrowth [36]. ATF3 inhibits fibroblast apoptosis through the TGF-β/Smad pathway [75]. In TGF-β1-induced synovial fibroblasts, ATF3 binds to the *RGS1* promoter, enhancing RGS1 expression, accelerating cell proliferation, and blocking apoptosis [76]. ATF3 plays a crucial role in injuries induced by ischemia/reperfusion [77,78]. In renal ischemia–reperfusion injury, overexpression of ATF3 protects HK2 cells from H_2_O_2_-induced cell death by inhibiting p53 and enhancing the expression of p21 [79]. The protein kinase p21 is involved in cellular apoptosis through its regulation of the cell cycle process [80]. Concurrently, p53 possesses the capacity to promote apoptosis in cells [81]. Research has reported that ATF3 inhibits the transcription of p53 by binding to the PF-1 site, subsequently suppressing cardiomyocyte apoptosis induced by doxorubicin, thereby exerting a protective effect on cardiomyocytes [74]. Thus, the inhibition of p53 transcription by ATF3 is a vital pathway in its anti-apoptotic function. In a study on brain injury following transient focal cerebral ischemia, *ATF3* knockout mice exhibited significantly higher infarct volumes, worsened neurological functions, and upregulation of neuronal apoptosis, inflammatory gene expression, and cellular inflammatory responses [82]. This indicates that ATF3 may be an essential protective regulatory factor in cerebral ischemic injury. Additionally, ATF3 can inhibit apoptosis mediated by cerebral ischemic injury through the downregulation of carboxy-terminal modulatory protein (CTMP), a pro-apoptotic factor that inhibits the anti-apoptotic Akt/PKB cascade [83].

These findings suggest a role for ATF3 in inhibiting apoptosis. However, ectopic expression of ATF3 improves the apoptotic capacity of topotecan-induced HeLa cells or camptothecin-induced HeLa cells [84]. ATF3 may act as a downstream target of the NF-κB and JNK/SAPK signaling pathways, promoting β-cell apoptosis [73]. ATF3 expression intensifies t-butyl hydroperoxide (TBHP)-induced apoptosis in nucleus pulposus cells (NPC) [85]. ATF3-dependent induction of RIPK3 causes a shift from apoptosis to necroptosis in hepatocytes [86]. The opposing regulation of DR5 and Bcl-xL expression by ATF3 promotes arsenic-induced apoptosis [87]. Forkhead transcription factors (FOXO3a) are a key molecule that promotes apoptosis, primarily functioning by facilitating the transcription of apoptosis-related factors, thereby mediating cell apoptosis [88,89,90,91]. The PI3K/Akt pathway inhibits apoptosis by phosphorylating FOXO3a, which prevents its nuclear translocation [90,91,92]. *Chelidonium majus* induces apoptosis in SKOV-3 cells by increasing the expression levels of ATF3 and its downstream protein, Tip60 [93]. Further studies have found that under *Chelidonium majus* stimulation, ATF3 enhances the expression of Tip60, which then promotes the dephosphorylation and nuclear translocation of FOXO3a, leading to apoptosis [93]. Tip60 is reported to acetylate p53 at K120, promoting the expression of the p53 target gene *PUMA*, thereby inducing apoptosis [94,95]. Research also reports that p53 promotes apoptosis by inhibiting the phosphorylation of PI3K/Akt [96]. Notably, it has been reported that Tip60 expression may be inversely correlated with PI3K/Akt activation [97,98]. Consequently, this may indicate that Tip60 acetylates p53, which in turn inhibits the phosphorylation of PI3K/Akt, subsequently leading to the dephosphorylation of FOXO3a. This sequence of molecular events mediates cellular apoptosis. This evidence highlights the role of ATF3 in promoting apoptosis.

### 6.2. The Regulation of Ferroptosis

**Figure 3:**
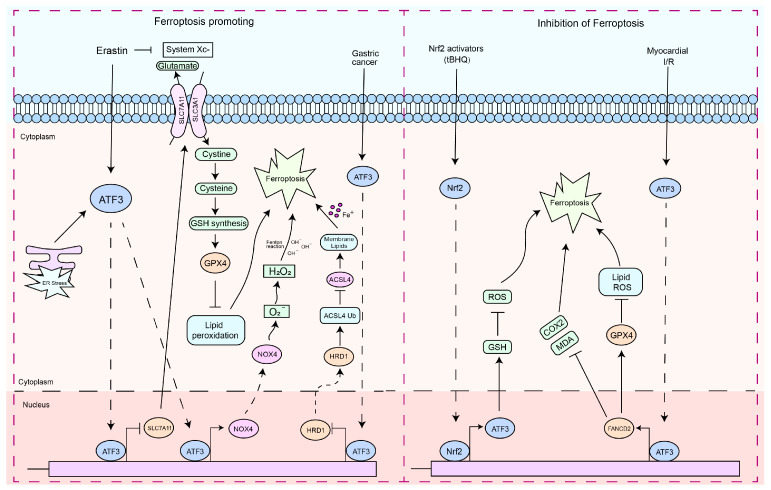
The functions of ATF3 in the ferroptosis response. These signaling pathways by which ATF3 regulates the ferroptosis response can be divided into two types: (1) pro-ferroptosis response—ATF3 triggers the ferroptosis pathway through the downregulation of SLC7A11. Stress from erastin and ER can induce ATF3 expression. ATF3 then binds to the promoters of the *SLC7A11* genes, downregulating their expression. ATF3 suppressed the Xc^−^, depleted the intracellular GSH, and thus promoted erastin-induced lipid peroxidation. ATF3 regulates ferroptosis by mediating transcription inhibition of GPX4 and HRD1. ATF3 contributes to cell ferroptosis by increasing NOX4 and H_2_O_2_. (2) Nrf2-dependent ATF3 contributes to the antioxidant functions of Nrf2. ATF3 inhibits ferroptosis by regulating the expression of FANCD2. Solid arrows indicate promotion, dashed arrows denote translocation, and horizontal arrows represent inhibition.

Ferroptosis is a nonapoptotic form of cell death that can be induced by metabolic stress, such as glutathione (GSH) depletion [28,99,100]. Recently defined as a newly discovered form of cell death, ferroptosis is different from apoptosis in that it does not involve caspase activation [99]. Ferroptosis leads to an increase in ROS and malondialdehyde (MDA), which ultimately causes overwhelming lipid peroxidation and results in cell death [99,101,102]. ATF3 is often involved in vital cellular activities such as metabolism. Currently, a large amount of research data indicates that ATF3 plays a significant role in the regulation of ferroptosis [28,31,102,103,104,105,106,107]. Nuclear factor erythroid 2 related factor 2 (Nrf2) can promote the expression of SLC7A11 and GPX4 under oxidative stress, which is crucial to mediate the onset of ferroptosis [108,109,110]. As an endogenous inhibitor of SLC7A11, ATF3 promotes erastin-induced ferroptosis by inhibiting the cystine/glutamate antiporter (system Xc-) [28]. Furthermore, ATF3 has been reported to facilitate ferroptosis in strychnine-induced glioblastoma cells by increasing H_2_O_2_ and suppressing SLC7A11 transcription [103]. In acute renal injury, an increase in ATF3 expression is observed, and ATF3 knockout markedly increases the expression of SLC7A11 and GPX4, which consequently improves the vitality of proximal tubular epithelial cells in the kidney [107]. ATF3 mediates osteoblast ferroptosis by inhibiting the expression of GPX4 and promoting the accumulation of lipid peroxides [105]. ATF3 induces ferroptosis in GC cells by transcriptionally repressing GPX4 and HRD1 [111]. HRD1 mediates the ubiquitination and degradation of ACSL4 to inhibit ferroptosis [111]. ACSL4 is a key molecule that promotes the ferroptosis process [112,113]. Interestingly, ATF3 also exhibits a dual role in the regulation of cellular ferroptosis (Figure 3). Nrf2 plays a key role in the defense system against oxidative stress [114]. To counter oxidative stress, the transcription factor Nrf2 binds to the antioxidant response element (ARE), mediating the transcriptional activation of its responsive genes and regulating the body’s defense mechanisms against oxidative damage [114,115]. Research has discovered that the promoter sequence of ATF3 contains ARE binding sites [26]. Furthermore, Nrf2 enhances its protective function against oxidative damage in astrocytes by upregulating the transcription of ATF3 [26]. ATF3 upregulates GSH levels to protect astrocytes from oxidative stress [26], and negatively regulates TLR4, which has been reported to promote ferroptosis [19,116,117]. ATF3 can inhibit the ferroptosis response induced by myocardial ischemia/reperfusion (I/R) by regulating the transcription of a series of ferroptosis-responsive genes, including *GPX4*, *Ptgs2*, *Fth1*, *FANCD2*, and *Nox1* [118]. GPX4 and FANCD2 are considered two important molecules for suppressing the ferroptotic response [119]. The absence of ATF3 leads to reduced expression of GPX4 during I/R and H/R injuries, while its re-expression upregulates GPX4 expression, accompanied by a reduction in Fe^2+^ accumulation, ROS production, and MDA release [118]. In erastin- and RSL3-treated cardiomyocytes, overexpression of ATF3 lowers the levels of Fe^2+^, ROS, and MDA associated with ferroptosis and reduces cell death [118]. This suggests that, in certain contexts, ATF3 can also act as an inhibitory factor for ferroptosis.

In general, the pathways of cellular apoptosis and ferroptosis are mediated by the downstream targets of ATF3. ATF3 mediates cell death by regulating the expression of genes related to apoptosis and ferroptosis, either directly or indirectly. However, the molecular mechanisms underlying the regulation of these cell death processes remain to be elucidated.

## 7. The Functions of ATF3 in the Pathogenic Microbial Infection Process

The interaction between hosts and pathogens represents a pivotal issue in the realm of infectious biology. During this intricate interplay, the response mechanisms of host cells are crucial for controlling infection and disease progression. In recent years, the function of ATF3 in the regulation of immune responses has been extensively studied, especially with regard to its expression and regulatory mechanism in host cells after pathogen infection. The expression of ATF3 can be induced by various pathogen infections, including bacteria and viruses (Table 1), indicating its universality on host defense mechanisms. Here, we summarize the role of ATF3 during pathogen infection and discuss its potential impact within host defense mechanisms.

### 7.1. The Functions of ATF3 in Viral Infection

Recent research has revealed the dual role of ATF3 in viral infections, where it can inhibit viral replication and can also be exploited by viruses to enhance their replication [120,121,122]. Therefore, the precise function of ATF3 depends on the specific host–virus interaction environment, as well as the type of infected cell and the species of virus involved.

#### 7.1.1. DNA Virus

The HBx protein, one of the seven proteins made by the Hepatitis B virus (HBV), is very toxic and can activate various genes in cells [122,123]. This affects many cell processes, such as the regulation of intracellular gene transcription, signal transduction, protein degradation, the cell cycle, and apoptosis [122,123]. Furthermore, HBx is a protein with a short half-life, primarily degraded in the cell via the ubiquitin-dependent proteasome pathway. Inhibiting HBx degradation and increasing the level of intracellular expression of HBx is one way HBV can cause liver cancer [123]. IL-1β/ATF3 promotes HBx mRNA degradation by mediating the expression of Ski2, which helps prevent complications mediated by HBx [122]. In particular, the level of Ski2 is also regulated by the HBx protein, forming a significant negative feedback loop to suppress HBx levels [122]. This mechanism is likely crucial for the virus to maintain an optimal concentration of HBx to support viral replication without triggering apoptosis [122,124]. Interestingly, HBx appears to induce *Ski2* promoter activity by interacting with ATF3 [121,122].

Murine cytomegalovirus (MCMV) is a virus particularly susceptible to control by IFN-γ, produced by NK cells. The viral load of MCMV in the liver is regulated by IFN-γ, which is secreted by NK cells in response to IL-12 [125,126,127]. Previous studies have shown that depletion of IFN-γ exacerbates infection, leading to an increase in liver MCMV viral load and the appearance of hepatitis [126,128,129]. Concurrently, IFN-γ derived from NK cells restricts MCMV replication and liver damage [126,130]. Mice with a knockout of the *ATF3* gene have been reported to exhibit a reduced liver viral load and less hepatic pathology after MCMV infection [126]. This is attributed to the role of ATF3 as a negative regulator of IFN-γ expression in NK cells rather than in T cells, thus facilitating MCMV infection [126].

A key characteristic of the Herpes Simplex Virus (HSV) is its ability to establish latent infection in the autonomic ganglia and reactivate under physical, hormonal, or emotional stress [131,132]. ATF3 is currently recognized as a significant stress-induced factor associated with the suppression of latent viral activation [132]. ATF3 has been reported to play a role in maintaining the latent state of HSV [132]. ATF3 is specifically expressed after neuronal injury, and it acts in synergy with STAT3 to induce the expression of downstream genes [133]. The main function of ATF3 in cells infected with HSV-1 has been reported to be to maintain neuronal integrity [132].

Human Papillomavirus (HPV) infection is a primary risk factor for cervical cancer [134,135]. HPV facilitates the proteolytic degradation and inactivation of p53 through the expression of the E6 protein [136,137]. Therefore, inhibiting the E6-promoted degradation of p53 appears to be an effective intervention against HPV-induced cervical cancer [137,138,139]. Research has found that ATF3 inhibits the E6-promoted degradation of p53 by directly binding to the HPV protein [138]. This interaction prevents the binding of E6AP to E6, thereby obstructing the recruitment of ubiquitin ligase to p53, reducing its ubiquitination and proteolytic degradation [138]. Unlike E6, ATF3 does not bind to E6AP, but it competes with E6AP to form a complex with E6 and p53 [137,138]. Further studies have found that ATF3 plays a significant role in inducing apoptosis in HeLa cells with depleted levels of p53 caused by HPV18 E6 activity [139]. They indicate that ATF3 plays a key role in a mechanism defending against HPV-induced carcinogenesis.

**Table 1:** The functions of ATF3 in the process of pathogenic microbial infections.

Microbial Name	Microbial Type	Functions of ATF3	Research Models	References
**Virus**				
Hepatitis B Virus	DNA virus	ATF3 increases HBx mRNA degradation by regulating Ski2 expression.	HepG2, PXB, and AML12 cells.	[121,122]
Murine CytoMegalovirus	DNA virus	ATF3 regulates anti-MCMV responses by controlling the production of IFN-γ in NK cells.	C57BL/6, *Rag1*−/−, BALB/c, and *ATF3*−/− mice.	[126]
Herpes Simplex Virus 1	DNA virus	ATF3 maintains the integrity of the neurons harboring latent virus.	HEp-2, Vero cell, HEK 293T, and CBA/J mice.	[132]
Human Papillomavirus	DNA virus	ATF3 plays a significant role in inducing apoptosis in HeLa cells.	HeLa cells.	[138,139]
Japanese Encephalitis Virus	RNA virus	ATF3 as a negative regulator of antiviral response and autophagy in mammalian cells during JEV infection.	Neuro2a, HEK, HeLa, and MEF cells.	[120]
Zika Virus	RNA virus	ATF3 acts to limit ZIKV infection by regulating autophagy and, thus, also ZIKV replication.	Wild-type and *ATF3* knockout A549 cell lines.	[140]
Coxsackievirus B3	RNA virus	-ATF3 regulates cell death induced by CVB3 infection.	HeLa cells.	[141]
Dengue Virus	RNA virus	Dengue virus degrades USP33-ATF3 axis via extracellular vesicles to activate microglial cells.	THP1 and HEK293T cells.	[142]
Human Immunodeficiency Virus	RNA virus	ATF3 orchestrates a recruitment of chromatin-modifying proteins.	Cervical carcinoma cell line C33A.	[143]
**Bacteria**				
*Staphylococcus aureus*	Gram-positive bacteria	ATF3 regulates antibacterial genes for antimicrobial processes.	Wild-type and *ATF3* knockout mice. RAW 264.7 cell lines.	[14,15]
*Streptococcus pneumoniae*	Gram-positive bacteria	ATF3 promotes cytokine production (IL-17A TNF-α, IL-1β, and IFN-γ) in response to *S. pneumoniae* infection.	C57BL/6 WT and *ATF3* KO mice. RAW 264.7 cells.	[4,13,17]
*Listeria monocytogenes*	Gram-positive bacteria	ATF3 provides protection from *L. monocytogenes* infections.	*ATF3* knockout and wild-type mice. A549, HEp2, and RAW 264.7 cells.	[15]
*Neisseria gonorrhoeae*	Gram-negative bacteria	ATF3 negatively regulates IL-6 expression during *N. gonorrhoeae* infection.	T84 colorectal epithelial cells, End 1 endocervical cells, nasopharyngeal cells, and bronchial epithelial cell line 16HBE14.	[144]
*Escherichia coli*	Gram-negative bacteria	ATF3-mediated suppression of the innate cytokine storm abrogated the control of bacteria and causes high susceptibility to secondary infections.	C57BL/6 WT and *ATF3* KO mice. A549, HEp2, and RAW 264.7 cells.	[15,53]
*Pseudomonas aeruginosa*	Gram-negative bacteria	ATF3 suppresses the progression of PA infection in hosts by inhibiting the activity of NF/κB.	AW264.7 and C57BL/6 *ATF3* KO mice.	[145,146]
*Mycobacterium tuberculosis*	Other bacteria	ATF3 promotes cell autophagy and suppresses inflammatory response in *Mycobacterium-tuberculosis*-infected A549 cells.	A549 cells and RAW264.7 cells. BALB/c mice.	[12,147]
*Mycoplasma pneumoniae*	Other bacteria	ATF3 inhibits the expression and release of TNF-α, IL-1β, IL-6, and IL-18 induced by *Mycoplasma pneumoniae* in vitro and in vivo.	BALB/c mice, C57BL/6 mice, and RAW264.7 cells.	[148]
**Fungi and Parasite**				
Patulin	Fungal toxin	Patulin enhances ATF3 expression and promotes apoptosis in colorectal cancer cells.	HCT116 cells.	[149]
Deoxynivalenol	Fungal toxin	Deoxynivalenol induces G2/M cell cycle arrest in HepG2 cells by ATF3ΔZip2a/2b.	HepG2 cells.	[150]
*Leishmania*	Parasite	ATF3 promotes the survival of the *Leishmania* by regulating inflammatory response.	RAW 264.7 and BMDM cells.	[151,152]

#### 7.1.2. RNA Virus

The binding of type I interferons (IFN) to their receptors leads to receptor dimerization, subsequently activating the IRF and STAT families of TF [120,153]. STAT1 and STAT2 undergo dimerization and interact with IRF9, resulting in the formation of the interferon-stimulated gene factor 3 (ISGF3) complex [120,153]. This complex then translocates to the nucleus and binds to the conserved interferon-stimulated response element, thus inducing a range of interferon-sensitive genes (ISGs) that inhibit the replication of the Japanese Encephalitis Virus (JEV) [120]. In addition to cellular antiviral signaling, autophagy has also been demonstrated to inhibit JEV replication [154]. ATF3 regulates viral infection by stimulating and inhibiting immune responses [20,140,155]. ATF3 has been reported to inhibit cellular antiviral signaling and autophagy by binding to the promoter regions of *STAT1*, *IRF9*, *ISG15*, and *ATG5*, thereby promoting JEV virus replication [120].

Interestingly, compared to its response to JEV infection, ATF3 exhibits a different function in hosts infected with the Zika virus (ZIKV). Research found that ATF3 inhibits ZIKV infection by differentially regulating the transcription of specific innate immune response and autophagy genes [140]. During the infection with ZIKV of A549 cells, ATF3 promotes the transcription of the *RIG-I*, *STAT1*, *IRF9*, and *ISG15* genes while simultaneously inhibiting the transcription levels of *IFNβ* and *IFIT2* [140]. In addition to modulating ER stress and innate immune responses, ZIKV also disrupts the autophagy pathway in the early stages of infection, thus facilitating viral replication [156,157]. ATF3 binds to the promoter sequences of the autophagy-related genes *Beclin-1* and *ATG5* [120,158] and inhibits their expression during ZIKV infection [140].

Viruses often need to inhibit host cell death in the early stages of infection to allow sufficient replication time for the production of adequate viral progeny [159]. Later in the infection, promoting host cell death or utilizing budding mechanisms can facilitate viral dissemination [159]. Research has found that after infecting HeLa cells, Coxsackievirus B3 (CVB3) regulates the downregulation of ATF3 to inhibit cell death, thereby ensuring enough replication time for generating sufficient viral progeny [141]. This suggests that the downregulation of ATF3 may act as a mechanism to attenuate cell death induced by CVB3 infection, enhancing the virus’s ability to infect the host.

Infection with the Dengue virus (DENV) can induce a robust cytokine storm in the brain, leading to neurological symptoms or death in the host [160]. An upregulation of ATF3 expression has been observed in blood samples of patients infected with DENV [161]. Research has discovered that monocytes infected with DENV secrete extracellular vesicles (EV), which are internalized by microglia [142]. The miR-148a carried within these EVs inhibits the expression level of the ubiquitin-specific peptidase 33 (USP33) protein. The reduction in USP33, in turn, decreases the stability of cellular ATF3 protein through deubiquitination, thereby promoting the expression of pro-inflammatory genes such as *TNF-α*, *NF-κB*, and *IFN-β* [142]. This indicates that DENV manipulates the EV pathway to transfer miR-148a, thereby regulating the levels of USP33 and downstream ATF3 in human microglia and leading to neuroinflammation within the central nervous system.

In the context of the pathogenesis of integrated viruses, a pivotal aspect involves the exploitation of host cellular machinery for the expression of the viral genome during host infection. Specifically, the viral genome’s integration into the host’s chromatin architecture necessitates a strategic utilization of the host’s gene regulatory systems [143,162]. This phenomenon is exemplified in the case of Human Immunodeficiency Virus Type 1 (HIV-1), where, post-infection, the viral genome becomes assimilated into the host genome as a component of chromatin [143,162]. Central to this process is Nuc-1, a nucleosome situated immediately downstream of the HIV-1 transcription initiation site, which inherently inhibits the activity of the long-terminal repeat (LTR) [143,162,163]. The initiation of LTR-driven transcription and consequent viral expression are contingent upon both epigenetic modifications and the disruption of nuc-1 [162]. Within this nucleosome, the presence of three AP1 sites is critical for the facilitation of viral transcription and replication [164,165,166]. Notably, the disruption of nuc-1 is rapidly induced following the treatment of latently infected cells with agents such as TNF-α or phorbol myristate acetate (PMA), both of which activate the AP1 and ATF/CREB pathways [163]. ATF3, a constituent of the ATF/CREB family, is instrumental in modulating gene translation. Some research suggests a potential role for ATF3 in HIV infection [143,167,168], postulating that the formation of a stable ATF3/JunB/HMGA1 complex at the periphery of nuc-1 orchestrates a sequential recruitment of chromatin-modifying enzymes, culminating in the disruption of nuc-1 [143]. These observations infer a broader regulatory capacity of ATF3, particularly in modulating the transcriptional expression of pathogenic microorganisms, with an emphasis on integrated viruses.

### 7.2. The Functions of ATF3 in Bacterial Infection

Cell death modes, inflammatory responses, and immune regulation play crucial roles in the interactions between bacteria and their hosts. ATF3 differentially regulates these physiological processes in both Gram-positive bacteria and Gram-negative bacteria.

#### 7.2.1. Gram-Positive Bacteria

During the infection process of *Staphylococcus aureus* (*S. aureus*), the host mediates the secretion of immune factors such as cytokines and chemokines through TLR-2, subsequently promoting the production of IL-17 to coordinate the host immune response [14,169]. IL-17, together with IL-22/IL-23, modulates macrophage function, thus inducing the expression of antimicrobial peptides (AMP) that kill or inactivate the pathogen [170,171]. Inactivation of IL-17, IL-22, and IL-23 leads to an increased *S. aureus load* and exacerbates the disease [170,172]. ATF3 has been reported to promote bacterial clearance by regulating the production of inflammatory cytokines, thus alleviating lethal *S. aureus* pneumonia [14]. ATF3 positively regulates the host’s resistance to *S. aureus* infection by modulating macrophage Reg3 expression and AMPs gene-mediated bacterial clearance, as well as the recruitment of macrophages, thus playing a significant role in the early stages of *S. aureus* infection [14].

IL-17A is critical in the early defense against *Streptococcus pneumoniae (S. pneumoniae)*, as mice lacking IL-17A and IL-17RA show increased vulnerability to bacterial pathogens that incite lung diseases [173,174,175]. ATF3 has been reported to facilitate the generation of IL-17A in γδ T cells through macrophage-mediated secretion of IL-1β, thus modulating the response to infection [4]. ATF3 regulates the immune response by maintaining the intracellular balance of ROS and calcium ions (Ca^2+^), influencing macrophage production of IL-1β and IL-23p19 [4]. This process is essential for stimulating the secretion of IL-17A, which is necessary for early defense against infections and crucial to eradicating *S. pneumoniae* [4]. Mice deficient in ATF3 exhibit reduced survival rates and an increased bacterial load in the lungs after infection with *S. pneumoniae* [4,13]. During infection, ATF3 actively adjusts innate immunity by enhancing the expression of TNF-α, IL-1β, and IFN-γ, thereby facilitating bacterial clearance [13]. The pneumococcal toxin pneumolysin (PLY) interacts with TLR4 to activate the MAPK pathway [17]. The activation of ATF3 depends on MAPK signaling. Upon activation, ATF3 enters the nucleus and interacts with c-Jun, promoting the production of cytokines, which in turn suppresses *S. pneumoniae* infection [17].

Wild-type mice demonstrate more effective bacterial clearance than ATF3-null mice during *Listeria monocytogenes (L. monocytogenes)* infection [15]. This suggests that ATF3 plays a critical role in resisting *L. monocytogenes* infection. PLY induces ATF3 expression through the TLR4/MAPK pathway [17]. Listeriolysin O (LLO), a member of the cytolysins released by *L. monocytogenes*, is known to stimulate TLR4-dependent cytokine expression and acts as a TLR4 agonist [176]. Furthermore, ATF3 significantly improves the expression levels of TNF-α, IL-1β, and IFN-γ during *L. monocytogenes* infection [15].

#### 7.2.2. Gram-Negative Bacteria

During episodes of bacterial sepsis, the host modulates its response to infection by upregulating or suppressing cytokines through ATF3 [13,53]. LPS significantly induces ATF3, which functions as a negative regulator in the production of inflammatory cytokines [19,53]. For example, in the context of *Escherichia coli* (*E. coli*) and *Neisseria gonorrhoeae* invasions, ATF3 acts as a negative regulator, suppressing the production of inflammatory cytokines [53,144]. ATF3 expression is stimulated in a MAPK-dependent manner during *Neisseria gonorrhoeae* infection [144]. In addition, the contraction of Tfp (Type IV pilus) further amplifies ATF3 expression. Subsequently, activated ATF3 then inhibits the transcription of the pro-inflammatory cytokine IL-6, thus promoting the progression of the infection [144].

*E. coli* sepsis is currently one of the most important types of sepsis [177]. ATF3 facilitates the progression of *E. coli* sepsis by suppressing IL-6 transcription [53]. Due to immunosuppression associated with ATF3-mediated sepsis, ATF3 knockout mice exhibit longer survival than wild-type mice after infection with *E. coli* [53]. Post-infection with uropathogenic *Escherichia coli* (UPEC), cytokines such as IL-1β, IL-6, and IFN-γ are significantly suppressed, while the bacterial load in the lungs and spleens of wild-type mice is substantially higher than in ATF3 knockout mice [15]. This indicates that ATF3 exhibits a divergent mechanism in Gram-positive and Gram-negative bacterial infections. This contradictory result may be caused by the following reasons: (1) LPS-induced ATF3 competes with NF-κB for binding to the promoters of target cytokines, thus inhibiting the production of inflammatory cytokines [15,19]. (2) LPS-induced ATF3 binds to cytokine promoters, and its interaction with HDAC leads to histone deacetylation. This process results in chromatin condensation, which suppresses cytokine gene transcription [15,19]. (3) The regulation of TLR4 differs; in Gram-positive bacterial infections, ATF3 may positively regulate TLR4 expression and stimulate cytokine production [13,15,17]. In contrast, LPS-induced activation of TLR4 enhances ATF3 expression, which acts to suppress the generation of inflammatory cytokines [19,20].

*Pseudomonas aeruginosa* (PA) induces acute lung injury in infected hosts through the release of inflammatory cytokines [178,179]. Thus, suppressing the host’s inflammatory response is crucial for resisting acute inflammation caused by PA. ATF3, a stress transcription factor known to regulate inflammatory responses, has also been reported to play a role in PA infection [145,146]. Research has found that ATF3 can bind with lipopolysaccharide-binding protein (LBP) to inhibit the activity of NF-κB and the inflammatory response, thereby protecting mice from acute lung injury induced by PA [146]. Another study indicates that during PA infection, ATF3 negatively regulates the translocation and Ser-536 phosphorylation of NF-κB p65, thus inhibiting the inflammatory response [145]. This suggests that ATF3 suppresses the progression of PA infection in hosts by inhibiting the activity of NF-κB.

In summary, the regulation of cytokines through the LPS/Toll/ATF3 axis has become a common phenomenon in Gram-negative bacteria.

#### 7.2.3. Other Bacteria

ATF3 expression is significantly upregulated during *Mycobacterium tuberculosis* (Mtb) infection [147]. ATF3 cooperates with BRG1 to activate the expression of the inflammatory cytokines IL-6, TNF-α, and IL-12p40 and increase the production of nitric oxide [12,147]. Foamy macrophages, a subpopulation of macrophages, play a pivotal role in the pathogenesis of tuberculosis [12]. They are characterized by an abundance of liposomes (LB), which may provide a survival environment for mycobacteria in granulomas [12,180]. Mtb stimulates the formation of LB-rich foamy macrophages [180,181]. ATF3 has been reported to inhibit liposome formation by regulating the expression of genes related to lipid metabolism [12]. This suggests that ATF3 limits Mtb survival by inhibiting LB formation.

ATF3 reduces the inflammatory response in the lungs of Mtb-infected mice by inhibiting IL-6 and IL-8 induced by the NF-κB pathway [147,182]. Mtb inhibits autophagy by impeding phagosome maturation [147,183,184]. Hence, facilitating the onset of autophagy is beneficial for the clearance of intracellular Mtb [147,183,184]. ATF3 has been reported to protect A549 cells from the inflammatory response induced by mycobacterial infection [12]. ATF3 induces autophagy and facilitates the clearance of mycobacteria by activating TIMP2 and inhibiting NF-κB [12].

Unlike Mtb, ATF3 inhibits cytokine production in macrophages infected with *Mycoplasma pneumoniae* (*M. pneumoniae*) [148]. Overexpression of ATF3 inhibits the expression and release of TNF-α, IL-1β, IL-6, and IL-18 from macrophages infected with *M. pneumoniae* [148]. Expression of ATF3 reduced the release of inflammatory cytokines in lung tissue and bronchoalveolar lavage (BALF) of mice infected with *M. pneumoniae*-infected mice [148]. This seems to imply that ATF3 also exhibits different roles in special pathogenic bacteria. This may be due to the fact that ATF3 plays different regulatory functions in response to different stimulation factors, different cell types, and different stress states. This undeniably enhances the intrigue surrounding research into the regulatory mechanisms of the ATF3 molecule.

### 7.3. The Functions of ATF3 in Fungal and Parasite Infections

Patulin is a fungal toxin primarily released by *Aspergillus* and *Penicillium* species [185]. Patulin exerts its toxic effect by covalently binding to reactive sulfhydryl groups in cellular proteins and by depleting glutathione, resulting in oxidative damage and the generation of reactive oxygen species (ROS) [186,187]. Research has found that Patulin induces transcription factor EGR-1 phosphorylation through increased oxidative stress, thereby enhancing ATF3 expression and promoting apoptosis in colorectal cancer cells [149]. Deoxynivalenol (DON), commonly known as vomitoxin, is a type B trichothecene mycotoxin predominantly produced by *Fusarium* species, such as *F. culmorum* and *F. graminearum* [188]. Interestingly, unlike Patulin, Deoxynivalenol induces G2/M cell cycle arrest in HepG2 cells by inducing the expression of EGR1 and p21 through the induction of ATF3’s splice variant, ATF3ΔZip2a/2b [150]. This once again highlights the complexity and significance of ATF3’s regulatory mechanisms. Notably, *Candida albicans*, a fungal pathogen that infects humans, significantly upregulates ATF3 after infecting cells [189,190]. This may suggest that ATF3 plays a role in *Candida albicans* infections.

The lifestyle of *Leishmania* is characterized by its role as an obligate intracellular pathogen, infecting the monocyte/macrophage lineage, which it enters through phagocytosis [191]. Consequently, *Leishmania* exhibits exceptional survival and replication capabilities in adverse environments [191]. Regulating the host’s anti-inflammatory environment and suppressing the production of superoxides are crucial for its persistent infection [192]. Research has found that ATF3 expression is upregulated in macrophages infected with *Leishmania* [151,152], and the survival rate of *Leishmania* in macrophages lacking ATF3 is reduced [152]. Studies have discovered that *Leishmania* upregulates the transcriptional activity of ATF3 via NRF2 [152]. Subsequently, ATF3 recruits HDAC1 to inhibit the transcriptional activity of NF-κB and IL-12b, thus promoting the survival of the parasite [152]. This suggests that *Leishmania* can manipulate the host’s inflammatory response through ATF3 to facilitate its own survival.

## 8. Prospects for Clinical Applications

Considering the multifaceted role of ATF3 in the regulation of physiological functions, it presents a broad prospect as a clinical pharmacological target: (1) Treatments that target ATF3 to control inflammation, useful in autoimmune and other inflammatory diseases. (2) Exploiting ATF3 to develop treatments for neurological disorders and nerve damage. (3) Utilizing ATF3 expression levels as a diagnostic and prognostic tool, especially in cancer. However, we need to carefully consider ATF3’s dual functions to avoid adverse effects in treatment. Targeting ATF3 presents significant opportunities for novel therapeutic developments and improved disease management in clinical medicine. Its potential as a drug target and a biomarker can lead to advances in personalized medicine. However, the complexity of ATF3’s roles necessitates careful research and development to ensure the efficacy and safety of these new approaches.

## 9. Conclusions and Future Perspectives

TFs are a significant class of protein factors involved in cellular activities that virtually participate in the regulation of all cellular life processes. ATF3, a stress-responsive transcription factor, is upregulated in response to cellular stressors such as oxidative stress, DNA damage, and inflammation, thus modulating the interactions between cellular metabolism, immunity, and inflammatory responses [193,194,195,196,197]. Consequently, ATF3 has emerged as a promising target for the treatment of certain inflammatory diseases. From our review of the literature, we conclude that ATF3 plays a complex and critical role in multiple biological processes. The function of ATF3 is dual, which is reflected in its response to inflammation, apoptosis, ferroptosis, and infection by pathogenic microorganisms. In terms of inflammation regulation, ATF3 has the ability to both promote and suppress inflammatory responses, with its specific action dependent on the type of stimulus encountered by the cell. This indicates that ATF3 is involved in the precise modulation of cellular responses to inflammatory signals. This dual functionality helps to maintain the balance of the immune system and prevent excessive inflammatory responses. In regulating apoptosis and ferroptosis, ATF3 similarly exhibits the capacity to promote and inhibit cell death. This underscores the key role of ATF3 in determining cell fate, further highlighting the diversity and complexity of ATF3 in cell death mechanisms. In response to pathogen infection, ATF3 can enhance or inhibit pathogen infection, potentially related to host cell defense mechanisms and pathogen evasion strategies. This finding suggests that ATF3 plays an important role in the development and prevention of infectious diseases.

Consequently, under the influence of varying cellular physiological states, ATF3 exhibits different regulatory functions. Furthermore, numerous questions merit further exploration. For example, why does ATF3 exhibit dual regulatory functions? Does ATF3 possess the capability to transition between its dual roles? What are the factors that induce this transformation? Is there an intrinsic link between ATF3 in inflammation, cell death, and responses to pathogenic microbial infections? For example, it is pertinent to investigate under which conditions ATF3-induced cell death is advantageous or detrimental to pathogen infection and propagation, or whether these processes trigger or inhibit more robust inflammatory immune responses. How pathogens exploit ATF3 to facilitate their replication and spread, or how host cells use ATF3-induced immune responses to limit the replication and dissemination of pathogens, are critical aspects warranting further research. As a key transcription factor in the host stress response, could ATF3 potentially influence the expression of pathogen-related genes, thereby regulating the process of pathogen infection? Therefore, the intricate internal balance among these factors deserves further study. Elucidating these mechanisms in detail could contribute to innovative breakthroughs in the prevention and treatment of inflammatory diseases and microbial infections.

## Data Availability

No data were used for the research described in the article.

## Supplementary Materials

The following supporting information can be downloaded at https://www.mdpi.com/article/10.3390/ijms25020824/s1.
