# Supplementary material for: The Dual Roles of Activating Transcription Factor 3 (ATF3) in Inflammation, Apoptosis, Ferroptosis, and Pathogen Infection Responses
Source: Int J Mol Sci. 2024 Jan 9;25(2):824. doi: 10.3390/ijms25020824 (PMC10815024; doi:10.3390/ijms25020824)
Supplement: Supplementary file 1 [file ijms-25-00824-s001.zip › ijms-2785386-supplementary.pdf]

Table S1 Motifs identified in the ATF3 binding sites

Gene	Motif (5'-3')	References
JunD	TGACGTCA	[18]
c-Jun	TGACGTCA	[18]
Nrf2	TGAGTCA	[18]
IL-6	CGACGTCA	[19]
IL-12b	GCATGATGTAA	[19]
AP1S2	TGGCGGCG	[47]
p53	TGACTCT	[74]
HSP27	TGACACTCT	[36]
RIPK3	GCTCCACCCCA	[86]
SLC7A11	TGATGCAAA	[28]
HRD1	AATGACTTAATT	[111]
